# DEPLOYR: a technical framework for deploying custom real-time machine learning models into the electronic medical record

**DOI:** 10.1093/jamia/ocad114

**Published:** 2023-06-27

**Authors:** Conor K Corbin, Rob Maclay, Aakash Acharya, Sreedevi Mony, Soumya Punnathanam, Rahul Thapa, Nikesh Kotecha, Nigam H Shah, Jonathan H Chen

**Affiliations:** Department of Biomedical Data Science, Stanford, California, USA; Stanford Children’s Health, Palo Alto, California, USA; Stanford Health Care, Palo Alto, California, USA; Stanford Health Care, Palo Alto, California, USA; Stanford Health Care, Palo Alto, California, USA; Stanford Health Care, Palo Alto, California, USA; Stanford Health Care, Palo Alto, California, USA; Center for Biomedical Informatics Research, Division of Hospital Medicine, Department of Medicine, Stanford University, School of Medicine, Stanford, California, USA; Center for Biomedical Informatics Research, Division of Hospital Medicine, Department of Medicine, Stanford University, School of Medicine, Stanford, California, USA

**Keywords:** machine learning, artificial intelligence, organizational readiness, computational infrastructure, healthcare organizations, clinical decision support

## Abstract

**Objective:**

Heatlhcare institutions are establishing frameworks to govern and promote the implementation of accurate, actionable, and reliable machine learning models that integrate with clinical workflow. Such governance frameworks require an accompanying technical framework to deploy models in a resource efficient, safe and high-quality manner. Here we present DEPLOYR, a technical framework for enabling real-time deployment and monitoring of researcher-created models into a widely used electronic medical record system.

**Materials and Methods:**

We discuss core functionality and design decisions, including mechanisms to trigger inference based on actions within electronic medical record software, modules that collect real-time data to make inferences, mechanisms that close-the-loop by displaying inferences back to end-users within their workflow, monitoring modules that track performance of deployed models over time, silent deployment capabilities, and mechanisms to prospectively evaluate a deployed model’s impact.

**Results:**

We demonstrate the use of DEPLOYR by silently deploying and prospectively evaluating 12 machine learning models trained using electronic medical record data that predict laboratory diagnostic results, triggered by clinician button-clicks in Stanford Health Care’s electronic medical record.

**Discussion:**

Our study highlights the need and feasibility for such silent deployment, because prospectively measured performance varies from retrospective estimates. When possible, we recommend using prospectively estimated performance measures during silent trials to make final go decisions for model deployment.

**Conclusion:**

Machine learning applications in healthcare are extensively researched, but successful translations to the bedside are rare. By describing DEPLOYR, we aim to inform machine learning deployment best practices and help bridge the model implementation gap.

## BACKGROUND AND SIGNIFICANCE

Access to real-world data streams like electronic medical records (EMRs) accelerates the promise of machine learning (ML) in healthcare. Over 250 000 studies exist related to risk-stratification models alone, many published in the past 10 years.^1^^,^^2^ Despite the hype, a sizeable gap separates ML models in research articles from those impacting clinical care.^3^^,^^4^ This implementation gap leaves most published clinical ML applications lost in the “model graveyard”.^5^

The gap between research and implementation demonstrates that strong predictive performance alone is not sufficient for feasible and worthwhile translation of clinical ML models to the bedside. Beyond demonstrating predictive accuracy, model champions must articulate actions that can be taken as a result of inferences (model outputs).^6^ Actions must incur utility, some measurably positive effect on a clinical outcome of interest benefiting the deployment population.^7–9^ Positive impact on average, however, does not imply positive impact for all. ML applications to healthcare must satisfy fairness principles, ensuring performance and accrued utility are not unfavorable across certain patient populations, particularly those traditionally under-served.^10–12^ Institutions leading the charge in the translation of clinical ML applications are establishing governance frameworks ensuring models are safe, reliable and useful throughout their deployment life-cycles.^13–17^

Even if the above are addressed, ML applications must overcome technical feasibility hurdles related to their deployment. Traditionally, if such technical implementations were possible at all within the context of existing vendor and legacy infrastructure, massive overhead was required to establish and maintain computational frameworks enabling ML deployment and integration into the EMR.^18–20^ Approaches of early-adopting institutions can be broken down into 2 broad categories.^21^ Some institutions rely on platforms native to their EMR vendor (eg, Epic Nebula) to deploy ML applications.^22^^,^^23^ These frameworks reduce maintenance overhead and facilitate model sharing across hospitals that use the same EMR.^24^^,^^25^ But deployment of researcher-developed models trained using an institution’s clinical data warehouse remains difficult, leading other institutions to develop custom frameworks. These solutions often tap into daily refreshes of EMR data (eg, from Epic Clarity) at inference time that do not support real-time use-cases.^21^^,^^26^ Successful solutions largely spawn from collaborations of data scientists who build and validate models and hospital information technology (IT) personnel who know the ins-and-outs of their institution’s EMR. These combinations of expertise are valuable but rare. Custom implementations allow flexibility out-of-the-box EMR vendor solutions do not, but design details largely remain internal knowledge, leaving other institutions to either re-invent the wheel or be left behind. This has led to a sparse and eclectic set of solutions with limited academic discourse of best practices.

## OBJECTIVE

Through a collaboration of data scientists at the Stanford School of Medicine, and IT persona at both Stanford Health Care and Stanford Children’s Health, our objective was to develop and describe DEPLOYR—a technical framework for deploying researcher created clinical ML applications directly into the EMR. The DEPLOYR framework does not rely on EMR vendor solutions like Epic Nebula, and supports real-time ML applications by tapping into an up-to-date data stream. In this article, we detail the mechanisms DEPLOYR enables and design decisions made during its formation to provide a blueprint for development and best practices for execution. Functions reviewed here include real-time closed-loop model trigger, data retrieval, inference, user-interface integration, and continuous monitoring. DEPLOYR enables enhanced prospective evaluations of ML models that extend beyond traditional retrospective evaluations. We demonstrate this capability and importance by silently deploying 12 previously retrospectively validated ML models triggered by clinician button-clicks in the EMR.

## MATERIALS AND METHODS

**Figure 1.**
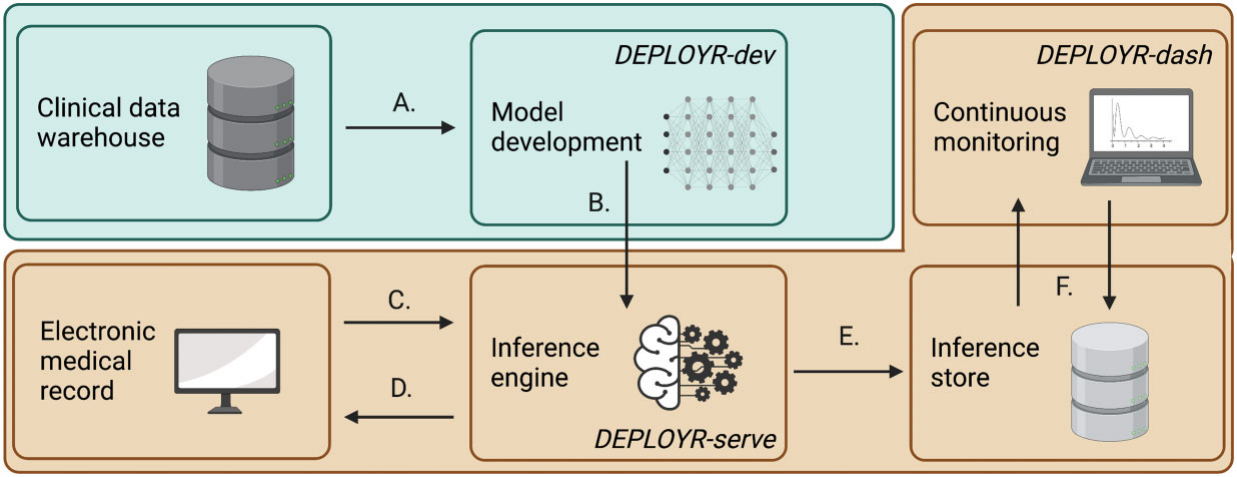
Summary of a DEPLOYR enabled model deployment. Blue shading indicates infrastructure operated by the Stanford School of Medicine (academic research). Orange shading indicates infrastructure operated by Stanford Health Care (clinical operations). (A) De-identified EMR data are sourced from Stanford’s clinical data warehouse (STARR), a model is developed and retrospectively validated using the *DEPLOYR-dev* python package. (B) The model is deployed to the inference engine, and exposed as a REST API using *DEPLOYR-serve*. (C) Inference is triggered, spawning an HTTPS request from the EMR directed at the exposed model. (D) The request results in the collection of a feature vector from the EMR’s transactional database using REST (both FHIR and EMR specific) APIs maintained by the EMR vendor. Inference is performed on the real-time retrieved data, and routed back to the EMR closing the loop with end-users and integrating into workflow. (E) Inferences and relevant metadata are additionally saved to the inference store, (F) and consumed by monitoring software (*DEPLOYR-dash*) that continuously tracks model performance via dashboard. REST: representation state transfer; HTTPS: hypertext transfer protocol secure; FHIR: fast healthcare interoperability resources.

Core functions of the DEPLOYR framework include data sourcing, inference triggers, and EMR integration. We detail our implementation of a monitoring module that tracks performance of deployed models over time, mechanisms that enable silent trial deployments, and prospective evaluations of model impact. DEPLOYR enables these mechanisms by leveraging integration capabilities native to Stanford Health Care’s EMR vendor (Epic Systems) in conjunction with 3 software applications: *DEPLOYR-dev* (a python package used for model development and validation), *DEPLOYR-serve* (a python Azure Function application to expose trained models as APIs), and *DEPLOYR-dash* (a dashboard implemented using the streamlit python package).^27^^,^^28^  Figure 1 provides a system level overview of DEPLOYR.

### Data sources

The design of ML deployment frameworks requires data sourcing decisions. To effectively translate research models, training data should be sourced from research-grade clinical data warehouses. At inference time, many ML use-cases require real-time and up-to-date data streams.^16^^,^^29–31^ Since clinical data warehouses are typically several transformations removed from these data streams, mappings must be implemented to ensure models receive the same data elements in training and deployment environments.

#### Training data source

DEPLOYR uses data from Stanford’s clinical data warehouse (STARR) for training.^32^ STARR contains deidentified EMR data from over 2.4 million unique patients spanning 2009–2021 who have visited Stanford Hospital (academic medical center in Palo Alto, CA), ValleyCare hospital (community hospital in Pleasanton, CA) and Stanford University Healthcare Alliance affiliated ambulatory clinics. STARR data are derived from the EMR through a series of ETLs (exchange transform load) developed and maintained by a research IT team within the school of medicine. Quality checks are executed after each transformation to mitigate error propagation and persistence.^32^ Use of STARR data was approved by the institutional review board of the Stanford University School of Medicine.

#### Inference data source

**Figure 2.**
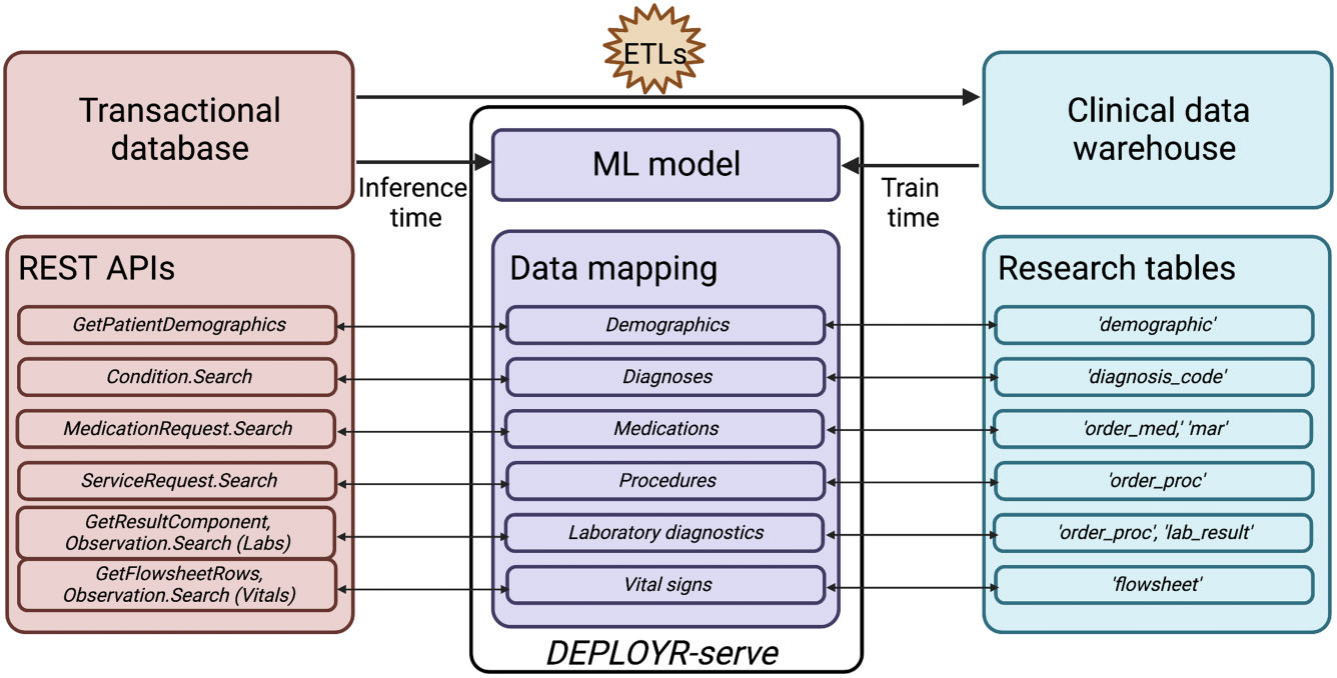
ML models are trained using data sourced from Stanford’s clinical data warehouse (STARR). In production, real-time data are sourced from the EMR’s transactional database (Epic Chronicles) through Epic and FHIR REST APIs. STARR data are several ETLs (extract, transform, loads) removed from the transactional database. Data mapping is necessary at inference time to ensure features seen during training match features seen in production. Mappings and inferences are invoked in *DEPLOYR-serve*.

In production, DEPLOYR sources data from the EMR’s transactional database, Epic Chronicles, which contains real-time patient data.^33^ We access this data stream using vendor specific and Fast Healthcare Interoperability Resources (FHIR) representational state transfer (REST) application programming interfaces (APIs) documented by our EMR vendor.^34^^,^^35^ Credentials enabling authentication to these APIs were provisioned upon registration of a back-end application to our EMR vendor’s application marketplace, Epic’s App Orchard. Use of this data source falls under the umbrella of hospital operational work specifically scoped to quality improvement. Data sources and mappings are depicted in Figure 2.

### Model inference triggers

Inference triggering mechanisms dictate how model’s can integrate into workflow. Additionally, because triggering logic specifies a model’s deployment population, it should be considered during cohort development to ensure the population in a researcher’s retrospective test set matches what is seen in production. DEPLOYR supports 2 classes of inference mechanisms—event- and time-based triggers.

#### Event-based triggers

Event-based triggers execute as a direct result of a clinical action. Examples include order entry for a laboratory diagnostic test, signature of a progress note, inpatient admission, or discharge. We enable event-based triggers by exposing models as REST APIs that listen for inbound HTTPS requests from the EMR. Models are wrapped in custom python functions using *DEPLOYR-serve*, an Azure Function application deployed to Stanford Health Care’s instance of Azure.^27^ Requests are spawned from the EMR through use of EMR alerts (eg, Epic Best Practice Advisories), rules, and programming points configured to execute upon button-clicks in the EMR’s graphical-user-interface, Epic Hyperspace.^36^^,^^37^ Requests from the EMR transmit patient identifiers to *DEPLOYR-serve* functions enabling patient specific feature vector collection.

#### Time-based triggers

**Figure 3.**
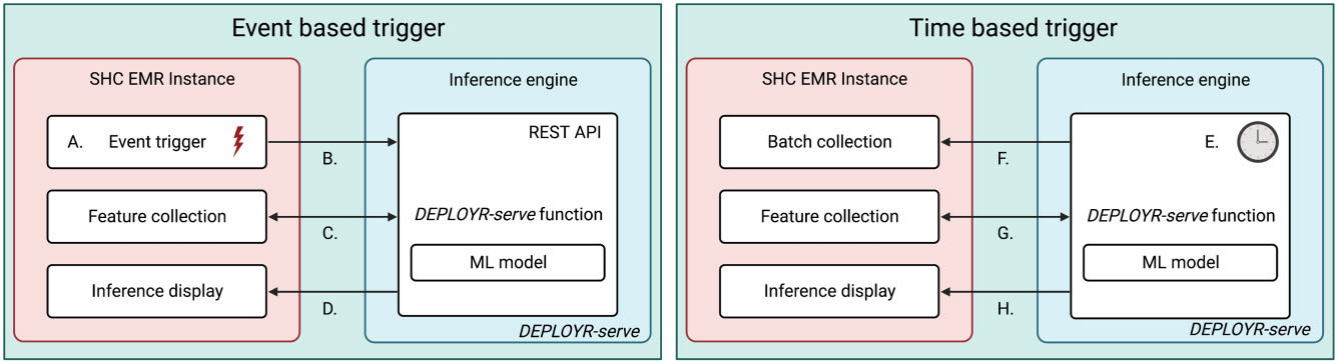
DEPLOYR triggering mechanisms. Models deployed with event-based triggering logic are exposed as REST APIs on the inference engine using a python Azure Function application (*DEPLOYR-serve*). An event (A) in the EMR (eg, clinician button-click initiating a laboratory order) transmits an HTTPS request (B) directed at the exposed *DEPLOYR-serve* function, which wraps an ML model. The function transmits HTTPS requests (C) to REST APIs documented in Epic’s App Orchard to collect a feature vector, performs model inference, and directs the inference and resulting clinical decision support via HTTPS request (D) back into the EMR to interface with end-users. Models deployed with time-based triggering logic perform inference at set intervals (E) through use of Azure Function timer triggers. Every time interval (eg, 15 min), a *DEPLOYR-serve* function transmits HTTPS requests (F) to REST APIs to retrieve a batch of patient identifiers for whom inference should be made. Feature vectors are collected for the batch of patients (G), and inferences are transmitted back into the EMR (H). SHC: Stanford Health Care.

Time-based triggers initiate inference periodically on batches of patients. Example applications include models that periodically monitor patients for deterioration, sepsis, or acute kidney injury.^38–41^  *DEPLOYR-serve* supports time-based triggering through use of Azure Function timer triggers configured using cron logic.^27^^,^^42^ Because time-based triggers do not originate from the EMR, DEPLOYR needs to determine which patients to perform inference on. *DEPLOYR-serve* functions select patients using vendor maintained APIs that collect patient identifiers, for example, within specific hospital units. Event- and time-based triggers are summarized in Figure 3.

### Directing model outputs

Deployment frameworks require mechanisms to direct model inferences and recommendations back to end-users, closing the loop. Although possible to communicate inferences via third-party web or mobile application, DEPLOYR integrates inferences into the EMR, reducing technical overhead and best fitting clinical workflow. Integration with the EMR is possible through several mechanisms, implemented in *DEPLOYR-serve*.

#### Passive integration

Inferences can be written to the EMR without interrupting clinical workflow, for example to an external model score column visible in inpatient lists and outpatient schedules. In Epic, score columns can be configured to display probabilities, binary flags, feature values and their contributions to promote interpretability.^43^ Additionally, inferences can be written to flowsheet rows and dynamic data elements, for example Epic Smart Data Values, which can be used to trigger downstream clinical decision support.^44^ Inferences can also be directed at clinicians using the EMR’s internal messaging system, for example Epic in-basket messages. Inferences are written to the EMR using APIs exposed and documented by our EMR vendor.

#### Active integration

**Figure 4.**
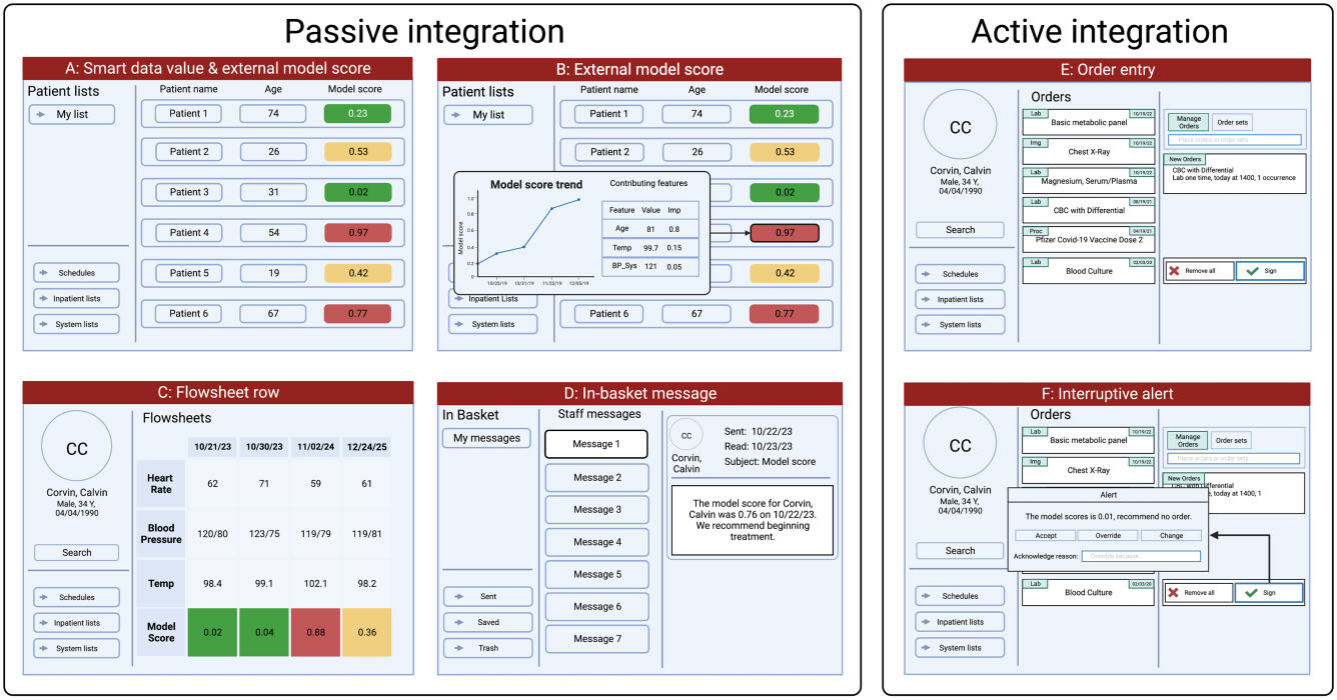
Mock-up frames depicting the EMR user-interface and mechanisms in which inferences (model outputs) can be displayed to end-users. Inferences can be directed back into the EMR passively, without interrupting clinical workflow. They can be written as smart data values or as external model score columns displayed in inpatient lists and outpatient schedules (A). Hovering the mouse over an external model score column value in a patient list displays a pop-up window (B) depicting the trend of the model score over time, feature values, and contribution of those features to the resulting inference. Model inferences can be written to flowsheet rows (C) and visualized over time in conjunction with other vital sign data (eg, heart rate, blood pressure, temperature). Inferences and suggested interventions can be directed as in-basket messages (D) to specific providers. Additionally, inferences can be integrated with the EMR actively through use of interruptive alerts (E, F) that trigger as a result of button-clicks (eg, signature of a laboratory order) in the user-interface. Inference integration is implemented in *DEPLOYR-serve*.

Some applications better integrate with workflow through interruptive alerts. Consider the ML use-case of flagging low-yield laboratory diagnostic testing to reduce wasteful ordering behavior.^31^ In production, inference could be triggered upon order entry of the diagnostic and interrupt the ordering process. Such active integration is possible through use of typical EMR alerts. Specifically, we use Epic Best Practice Advisory web-services. Epic supports 2 styles of web-services: classic clinical document architecture (CDA) and CDS web-hooks.^45^ DEPLOYR currently uses classic CDA web-services, which direct HTTPS requests at *DEPLOYR-serve* functions and await XML (Extensible Markup Language) responses.^46^ We show mock-ups of EMR integration capabilities in Figure 4.

### Continuous performance monitoring

Deployed models must be monitored to ensure continued reliability in production.^47–49^ All models are subject to potential performance decay over time due to distribution shift. Examples include covariate shift (changes over features), label shift (changes over labels), and concept shift (changes to the relationship between features and labels).^2^^,^^50^

#### Extracting labels in production

DEPLOYR uses *LabelExtactors* to collect labels in production. *LabelExtractors*, implemented in *DEPLOYR-serve*, are specific to a deployed model. They execute periodically using cron logic. At inference time, in addition to integrating model outputs to the EMR, we package inferences with relevant metadata (identifiers, timestamps, features) in inference packets and save them to the inference store, an Azure Cosmos database maintained by Stanford Health Care. *LabelExtactors* consume inference packets and pair to them their corresponding labels (once observable). A model tasked with predicting unplanned (30-day readmission) might produce inference packets that include the patient’s FHIR identifier, discharge time, and inference. The corresponding *LabelExtractor* would use the FHIR identifier and discharge time to make requests directed at relevant APIs, determining whether the patient was readmitted to the hospital within 30 days of the produced inference.

#### Tracking model performance

After a *LabelExtractor* has collected labels, performance metrics can be estimated. When monitoring binary classifiers, metrics may include threshold dependent measures like accuracy, sensitivity, specificity, precision along with threshold independent metrics like area under the receiver operator characteristics curve (AUROC). Plots that track discrimination ability (ROC and precision-recall curves) and calibration (calibration curves) can be constructed. In addition to population estimates, metrics can be monitored over patient subgroups, including protected demographic classes. Beyond classic ML metrics, measures of model usefulness such as net benefit and expected utility can be tracked to ensure model use is yielding more good than harm.^7^^,^^51^

#### Tracking distribution shifts in features and labels

**Figure 5.**
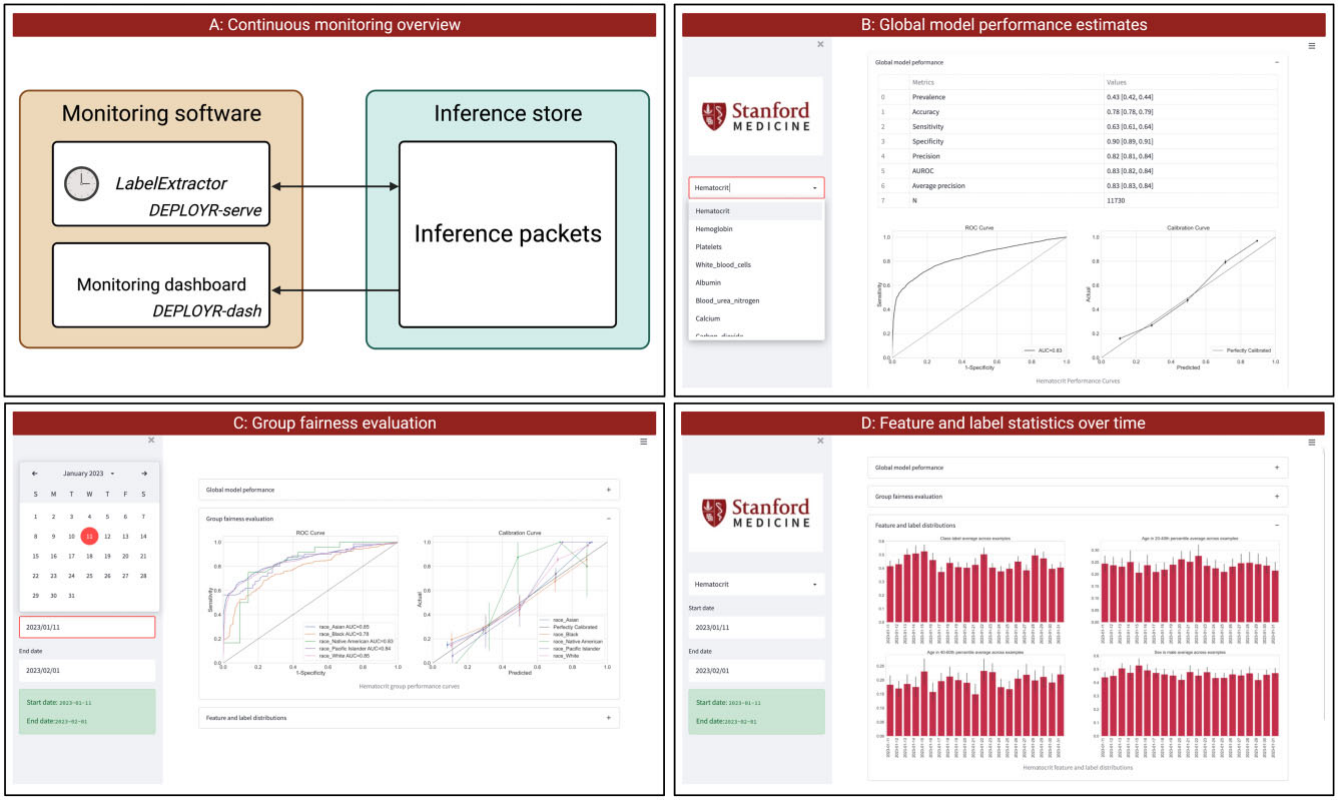
DEPLOYR performance monitoring. Panel A depicts the flow of data from monitoring software to the inference store. A *LabelExtactor* is implemented in *DEPLOYR-serve* as an Azure Function timer trigger. When executed it collects inference packets and pairs them with their corresponding labels. Inference packets paired with labels are consumed by a monitoring dashboard, implemented in *DEPLOYR-dash*. Panel B shows a screenshot of the dashboard displaying global model performance for the user-specified model. Panel C shows a group fairness evaluation across protected demographic classes for the user-specified time window. Panel D shows feature and label distribution statistics collected and tracked over time.

Performance decay over time is often attributable to distributional shift. Beyond calculating prospective performance metrics, DEPLOYR monitoring infrastructure tracks statistics that describe the distributions of features, labels, and predictions over time. Both model performance and distributional statistics are displayed to a dashboard created using the streamlit python package, which we show in Figure 5.^28^

### Enabling silent deployment

Deployment infrastructure requires the ability to silently test models prospectively.^14^^,^^52^^,^^53^ Silent trials allow data scientists to ensure data pipes are appropriately linked, and provide more robust appraisals of model performance than typical retrospective analyses because they occur directly in the intended production environment.^54^ Additionally, silent deployment can uncover faulty cohort design, for instance when a retrospective cohort was generated using exclusion criteria observable only after inference time, common when generating ML cohorts using case–control study design.^55^^,^^56^ DEPLOYR event- and time-based triggers can be configured to execute in the background, enabling silent trials.

### Enabling prospective evaluation of impact

The ultimate evaluation of healthcare ML impact is not a prospective ROC curve, but rather estimation of the causal effect of the model’s implementation on a clinical or operational outcome. In some use-cases, a model’s inference and corresponding recommendation will only be displayed to the end-user if predicted risk exceeds some threshold.^26^ Here, regression discontinuity designs may be suitable to estimate the local treatment effect of the model’s implementation.^57^ In other cases when an average treatment effect on the deployment population at large is desired, tracking end-user adherence to model suggestions (eg, whether an alert was accepted) can enable observational estimates of the intervention’s effect.^58^ These analyses require traditional assumptions from causal inference literature, for example no unobserved confounding, positivity, and consistency to produce unbiased effect estimates. Under equipoise, randomized study designs may be appropriate to achieve unbiased effect estimates.^59^^,^^60^ DEPLOYR supports functionality to inject randomization into the inference-directing mechanism to enable prospective determination of a model’s impact.

### Silent trial deployment of laboratory prediction models

To evaluate DEPLOYR, we silently deployed 12 binary classifiers that predict laboratory diagnostic results, building off of previous retrospective analyses of similar models designed to reduce wasteful laboratory utilization.^31^^,^^61–63^ Models were trained using STARR data, and exposed as REST APIs using *DEPLOYR-serve*. EMR alerts were configured to trigger the deployed models upon signature of the diagnostic test whose result each model aimed to predict.

#### Cohort and prediction task definitions

Three retrospective cohorts specific to a laboratory diagnostic exam were constructed. The unit of observation was an order for the exam. One cohort included orders for complete blood count (CBC) with differential diagnostics. Another included orders of metabolic panels. A third included orders for magnesium diagnostics. CBC and metabolic panel diagnostics result in several components, whereas magnesium exams yield a single result. Consistent with prior work, we trained binary classifiers per component to, at order time, predict whether the test result would fall outside the clinical laboratory defined normal reference range.^31^^,^^62^ Four binary classifiers were trained for the CBC cohort that separately predicted hematocrit, hemogloblin, white blood cell, and platelet results. Seven binary classifiers were trained for the metabolic panel cohort to predict albumin, blood urea nitrogen, calcium, carbon dioxide, creatinine, potassium, and sodium results. One classifier was trained to predict magnesium results. Retrospective cohorts spanned from 2015 to 2021, with 2000 diagnostic tests sampled randomly per year for a total of 14 000 orders per task. Corresponding prospective cohorts were collected in real-time during our silent deployment trial—spanning the dates January 11 to February 15, 2023.

#### Model training and evaluation

We trained and deployed random forest classifiers, as tree-based models are strong baselines for EMR-based ML tasks.^64^^,^^65^ A description of random forests as well as other model classes considered are detailed in Supplementary Note S1. Features included patient demographics, diagnosis codes mentioned on the problem list, medication orders, and prior lab results. Features were represented as counts, as detailed in Supplementary Note S2.^66^ All 12 classifiers were evaluated using retrospective and prospective test sets. We measured model discrimination ability by estimating the AUROC. The 95% confidence intervals were estimated by bootstrapping the corresponding test set 1000 times.

## RESULTS

Here we present the results of our retrospective and prospective silent trial evaluations. In Table 1, we summarize demographic characteristics of patients in both retrospective and prospective cohorts. In Table 2, we summarize model performance estimated using retrospective and prospective test sets. We compute prevalence of the positive class (an abnormal diagnostic result), as well as AUROC. Additionally, we constructed retrospective and prospective receiver operating characteristics (ROC) curves, precision recall (PR) curves, and calibration plots—shown in the Supplementary Figures S1–S3. We estimated model performance across patient subgroups stratified by protected demographic classes, which we show in Supplementary Tables S1–S12. Additionally, we show retrospective and prospective AUROC stratified by the number of available features in Supplementary Figure S4.

**Table 1.** Demographic breakdown of retrospective and prospective cohorts

Demographic breakdown		Retrospective cohorts			Prospective cohorts		
Diagnostic		CBC	Magnesium	Metabolic	CBC	Magnesium	Metabolic
*N* unique patients		13 362	11 771	13 410	18 982	5234	16 441
Age, mean (SD)		51.4 (23.7)	53.1 (24.2)	54.6 (21.4)	55.7 (21.4)	62.1 (17.8)	55.4 (21.1)
Sex, *n* (%)	Female	7103 (53.2)	5432 (46.1)	6923 (51.6)	10 092 (53.2)	2425 (46.3)	8711 (53.0)
	Male	6259 (46.8)	6338 (53.8)	6485 (48.4)	8884 (46.8)	2808 (53.6)	7724 (47.0)
	Unknown	0 (0.0)	1 (0.0)	2 (0.0)	6 (0.0)	1 (0.0)	6 (0.0)
Race, *n* (%)	White	6888 (51.5)	6038 (51.3)	6915 (51.6)	9009 (47.5)	2726 (52.1)	7636 (46.4)
	Other	2870 (21.5)	2899 (24.6)	2680 (20.0)	4545 (23.9)	1067 (20.4)	4103 (25.0)
	Asian	2444 (18.3)	1739 (14.8)	2600 (19.4)	3612 (19.0)	928 (17.7)	3125 (19.0)
	Black	557 (4.2)	587 (5.0)	583 (4.3)	1060 (5.6)	327 (6.2)	884 (5.4)
	Unknown	391 (2.9)	238 (2.0)	420 (3.1)	382 (2.0)	56 (1.1)	353 (2.1)
	Pacific Islander	168 (1.3)	212 (1.8)	171 (1.3)	294 (1.5)	98 (1.9)	261 (1.6)
	Native American	44 (0.3)	58 (0.5)	41 (0.3)	80 (0.4)	32 (0.6)	79 (0.5)

*Note*: Retrospective cohorts were sourced from Stanford’s clinical data warehouse (STARR). Prospective cohorts were collected in real-time through the EMR transactional database (Epic Chronicles) as diagnostic orders triggered model inferences between January 11 and February 15, 2023.

CBC: complete blood count.

**Table 2.** Model performance estimates on retrospective and prospectively collected test sets for all twelve models

Model performance		Positive (abnormal) prevalence		AUROC	
Diagnostic	Component	Retrospective	Prospective	Retrospective	Prospective
CBC with differential	Hematocrit	0.47 [0.45, 0.49]	0.43 [0.42, 0.44]	0.86 [0.85, 0.88]	0.83 [0.83, 0.84]
	Hemoglobin	0.50 [0.48, 0.52]	0.47 [0.46, 0.47]	0.88 [0.86, 0.89]	0.83 [0.83, 0.84]
	Platelets	0.26 [0.24, 0.28]	0.21 [0.21, 0.22]	0.79 [0.77, 0.82]	0.77 [0.76, 0.78]
	White blood cell	0.29 [0.27, 0.31]	0.27 [0.27, 0.28]	0.76 [0.74, 0.79]	0.69 [0.68, 0.70]
Metabolic panel	Albumin	0.20 [0.18, 0.21]	0.21 [0.20, 0.21]	0.88 [0.86, 0.91]	0.85 [0.84, 0.86]
	Blood urea nitrogen	0.22 [0.20, 0.24]	0.24 [0.23, 0.25]	0.85 [0.83, 0.87]	0.80 [0.79, 0.81]
	Calcium	0.11 [0.10, 0.13]	0.11 [0.11, 0.12]	0.80 [0.76, 0.83]	0.79 [0.78, 0.81]
	Carbon dioxide	0.16 [0.14, 0.17]	0.20 [0.20, 0.21]	0.69 [0.66, 0.72]	0.62 [0.61, 0.63]
	Creatinine	0.31 [0.29, 0.33]	0.32 [0.31, 0.33]	0.78 [0.75, 0.80]	0.75 [0.74, 0.76]
	Potassium	0.06 [0.05, 0.08]	0.08 [0.08, 0.09]	0.67 [0.61, 0.72]	0.60 [0.59, 0.62]
	Sodium	0.12 [0.10, 0.13]	0.17 [0.16, 0.17]	0.79 [0.75, 0.82]	0.71 [0.70, 0.72]
Magnesium	Magnesium	0.15 [0.14, 0.17]	0.14 [0.13, 0.15]	0.70 [0.67, 0.73]	0.65 [0.63, 0.67]

AUROC: area under the receiver operating characteristics curve; CBC: complete blood count.

## DISCUSSION

Our silent trial results underscore the necessity of prospective evaluations before ML applications are integrated into clinical work-streams.^14^ Across our 12 laboratory predictions tasks, AUROC in our prospective test sets were generally several percentage points lower than what is seen retrospectively. Drops in performance were similarly apparent in our ROC and PR curves, though the shapes of the curves remained similar across settings. Poorer performance could be attributable to data drift, data-elements appearing to be accessible at inference time in the clinical data warehouse not actually being accessible in production, and imperfect mappings between training and inference data sources. In Supplementary Figure S4 we show model performance stratified by number of available features at inference time, which differs between retrospective and prospective settings. Due to these deviations, we recommend using performance measures estimated from prospectively collected test sets during silent trials to make final go decisions when deploying clinical ML models.

In our evaluation we silently deploy 12 random forest classifiers, but DEPLOYR allows integration of arbitrary model classes ranging from simple regressions to complicated neural networks.^67^^,^^68^ This feature is attributable to DEPLOYR’s use of server-less compute, which is separate from the EMR and allows users to expose arbitrary code as REST endpoints.^22^^,^^25^ While DEPLOYR is model class agnostic, latency will increase with increasing model complexity. If latency is an issue, data scientists should consider whether the more complicated model is necessary to achieve desired model performance, and if so serve the model using a graphics processing unit (GPU), which server-less functions support.^69^

Continuous monitoring tools are essential to any deployment framework.^47^ Statistically significant and clinically meaningful variations in performance can indicate models need re-training or decommissioning.^70–72^ Careful attention to feedback mechanisms from interventions administered as a result of model integration must be considered. Feedback mechanisms occur when predictions lead to interventions that alter the distribution from which prospective data are generated. When monitoring risk-stratification models, interventions assigned to high risk patients intending to reduce risk can make models appear as “victims of their own success”.^73^ Models intending to discourage diagnostics test orders whose results are highly predictable, for example those we silently evaluate in this study, will induce censoring of the most predictable labels. Feedback mechanisms can cause drastic deviations between observed and actual performance.^73–75^ Naively updating these models do more harm than good.^76^ DEPLOYR supports injecting randomization into inference integration, which can be used in tandem with traditional weighting estimators to recover true performance in the presence of feedback mechanisms.^77^ The ability to randomize is similarly critical for deliberate randomized controlled trial evaluations of ML model impact on clinical outcomes.^59^^,^^60^

DEPLOYR can serve and evaluate a broad class of models for a range of clinical applications. Though we deploy binary classifiers in this study, the framework naturally extends to multi-class, multi-label and regression settings, which may be more suitable for alternative clinical applications including emergency department triage, recommender systems, and insulin dose optimization.^78–80^ Performance metrics a data scientist chooses to monitor will vary depending on the task. In a binary setting, a data scientist might monitor an ROC curve, in a multi-class setting a confusion matrix, and in a regression setting mean-squared-error. Irrespective of the exact metric set, the overarching goals of continuously monitoring remain the same. Because DEPLOYR leverages server-less compute that allows execution of arbitrary software, data scientists have control over postprocessing of inferences and the metrics they choose monitor in deployment.

The DEPLOYR framework can be extended to institutions that (1) have access to a clinical data warehouse for model development and (2) maintain 21st Century Cures Act compliant FHIR APIs.^81^^,^^82^ Though the DEPLOYR code base does also utilize Epic specific APIs, making extensions to institutions that vendor with Epic Systems easier, similar FHIR-based APIs exist that serve as suitable alternatives.^83^ Additionally, while our installation of DEPLOYR uses an EMR alerting mechanism native to Epic Systems to trigger model inferences (Epic Best Practice Advisories), similar alerting mechanisms are maintained by alternative EMRs that would enable equivalent functionality.^84^^,^^85^

Successful installation and maintenance of DEPLOYR requires dedicated financial resources and personnel. DEPLOYR’s computing infrastructure is resource efficient. The total cloud computing cost for our silent deployment which lasted between January 11 and February 15, 2023 (Azure Function executions and data storage in Azure Cosmos) was less than $300.^86^^,^^87^ To assign dedicated personnel, Stanford Health Care’s IT department has formed a data science team. Expertise in engineering is required to adequately connect data pipes and manage cloud infrastructure. Data scientists are required to develop, monitor and evaluate deployed models. EMR integration expertise is required to set up EMR alerts, ensure the EMR can communicate with the server-less function application, and maintain integrations in the advent of EMR upgrades. Networking and security personnel are required to ensure data are appropriately routed between the EMR and the institution’s cloud infrastructure. At Stanford Health Care, data never leaves our internal network.

Limitations to this study include that, while the DEPLOYR framework is general and can be extended to additional institutions that use alternative EMRs, expertise and support from an institution’s IT department would be required to translate the Epic specific integrations we leverage (eg, Best Practice Advisories) and data mappings to alternative EMR solutions and clinical data warehouse structures. The design decisions and considerations we have outlined in this article remain relevant to any institution attempting to install a deployment framework of their own. We note that EMR data are notoriously messy and frequently incomplete. Missing data can influence performance of clinical ML models, which we demonstrate in Supplementary Figure S4. We show that performance of our ML models increases with the number of available features at inference time. Data scientists tasked with deploying ML models may benefit from specifying a tolerance threshold on the sparsity of a feature vector used for inference, which may boost performance in production by restricting inferences to examples a model is more likely to correctly classify. While DEPLOYR can even include unstructured EMR data represented in clinical notes, future upgrades would be necessary to support a broader array of clinical ML applications that use multiple data modalities.^88–90^

## CONCLUSION

There exist frameworks to govern and promote the implementation of accurate, actionable and reliable models that integrate with clinical workflow.^14^^,^^15^ The creation of such frameworks has created the need for accompanying technical solutions that enable executing best practices laid out as desiderata.^53^ To enable adherence to such guidance, we have developed and demonstrated DEPLOYR, a technical framework for rapidly deploying as well as prospectively monitoring custom, researcher developed ML models trained using clinical data warehouses. The framework’s design elements and capabilities are demonstrated via silent deployment of multiple ML models triggered by button-clicks in Stanford Healthcare’s production EMR instance, enabling prospective evaluation of the models’ performance on unseen data directly in the deployment environment. The ability to perform enhanced prospective evaluations of researcher created ML models that extend beyond typical retrospective analyses supports the adoption of best practices that can close the ML implementation gap.

## Supplementary Material

ocad114_Supplementary_Data

## Data Availability

De-identified electronic medical record data used here for model training is made available through STARR, STAnford medicine Research data Repository.^32^ The data can be accessed for research purposes after Institutional Review Board approval via the Stanford Research Informatics Center.

## CODE AVAILABILITY

The DEPLOYR framework uses 3 distinct software repositories, *DEPLOYR-dev* to train and validate models, *DEPLOYR-serve* to expose models to the inference engine and *DEPLOYR-dash* to monitor and visualize prospective model performance characteristics. While this software is specifically designed to interact with Stanford data models and systems, we make *DEPLOYR-dev*, *DEPLOYR-dash* and an example of *DEPLOYR-serve* publicly available for reference.

- *DEPLOYR-dev:*  https://github.com/HealthRex/deployr-dev
- *DEPLOYR-serve:*  https://github.com/HealthRex/deployr-serve
- *DEPLOYR-dash:*  https://github.com/HealthRex/deployr-dash
