# Supplementary material for: DEPLOYR: a technical framework for deploying custom real-time machine learning models into the electronic medical record
Source: J Am Med Inform Assoc. 2023 Jun 27;30(9):1532–42. doi: 10.1093/jamia/ocad114 (PMC10436147; doi:10.1093/jamia/ocad114)
Supplement: ocad114_Supplementary_Data [file ocad114_supplementary_data.pdf]

Supplementary Materials

Supplemental Note 1

Machine learning model classes considered for our prediction tasks included logistic regressions with L1 (lasso) and L2 (ridge) penalties, random forest classifiers, and gradient boosted trees. L1 and L2 penalized logistic regressions are suitable for wide, sparse and collinear data, like electronic medical record data, as the penalty terms act to perform feature selection intrinsically within their fitting procedures. While these model classes lack flexibility that non-linear tree based models provide, they are frequently used in situations where it is desirable to prevent overfitting. Random forests and gradient boosted tree model classes model non-linearities within the data, and thus provide flexibility to fit more arbitrary functions. Beyond flexibility, tree based models naturally handle feature selection in the presence of wide, sparse and collinear data, making them desirable model classes for electronic medical record machine learning tasks. While tree based models have increased flexibility compared to L1 and L2 logistic regressions, they are also more prone to overfit to the provided training set. This can be mitigated in random forest models by increasing the minimum number of samples allowed in a leaf node, and reducing the max depth of each tree. Overfitting in gradient boosted trees can be prevented by using shallower trees, and by using an early-stopping criteria on the number of boosting rounds through use of an additional validation set. Consistent with best practice, we selected random forest models for final evaluation across our twelve machine learning tasks because they performed best on our retrospective validation sets.

Supplemental Note 2

We created feature matrices for each cohort using count based representations. For each cohort, a timeline of medical events was constructed from structured electronic medical record data available before inference time. A mix of categorical and numerical data elements were considered. Categorical features included diagnosis (ICD 10) codes on a patient's problem list, medication orders and demographic variables including race and sex. Numerical features included prior laboratory results and the patient's age at inference time. Numerical features were discretized into tokens based on the percentile values they assumed in the training set distribution. All numerical features were binned into five buckets. All diagnosis codes prior to prediction time were included in the patient's constructed timeline. Medication orders placed within 28 days of prediction time were included, as were laboratory results made available within 14 days of prediction time. Sequences of tokens were then transformed into feature vectors in bag of words (counts) fashion. The total number of features was 14145, 13631, and 14917 for the CBC, metabolic panel, and magnesium cohorts respectively. Each cohort was split into training, validation and test sets based on the year the diagnostic order took place. Training sets included the years 2015 to 2019. Validation sets included orders taking place in 2020, and test sets included orders taking place in 2021. Model's were trained using the training sets, hyperparameters and final model class were chosen using the validation sets, and performance evaluated on the test sets. Though we deployed random forests using the sklearn python package, DEPLOYR allows any arbitrary model class or package.

Supplementary Table 1: Hematocrit model performance by protected demographic groups

Prediction task	Group	Retrospective AUROC	Prospective AUROC
**Hematocrit**	***Full cohort***	***0.86 [0.85, 0.88]***	***0.83 [0.83, 0.84]***
	sex_Female	0.85 [0.83, 0.88]	0.83 [0.82, 0.84]
	sex_Male	0.87 [0.84, 0.89]	0.83 [0.83, 0.84]
	race_Asian	0.85 [0.81, 0.88]	0.85 [0.83, 0.86]
	race_Black	0.84 [0.75, 0.91]	0.79 [0.76, 0.81]
	race_Native American	0.87 [0.55, 1.00]	0.81 [0.70, 0.90]
	race_Other	0.85 [0.81, 0.89]	0.82 [0.81, 0.83]
	race_Pacific Islander	0.91 [0.70, 1.00]	0.85 [0.81, 0.89]
	race_Unknown	0.80 [0.66, 0.90]	0.75 [0.68, 0.81]
	race_White	0.88 [0.85, 0.90]	0.84 [0.83, 0.85]
	age_over_40	0.87 [0.85, 0.89]	0.84 [0.83, 0.85]
	sex_Unknown	NaN	1.00 [1.00, 1.00]

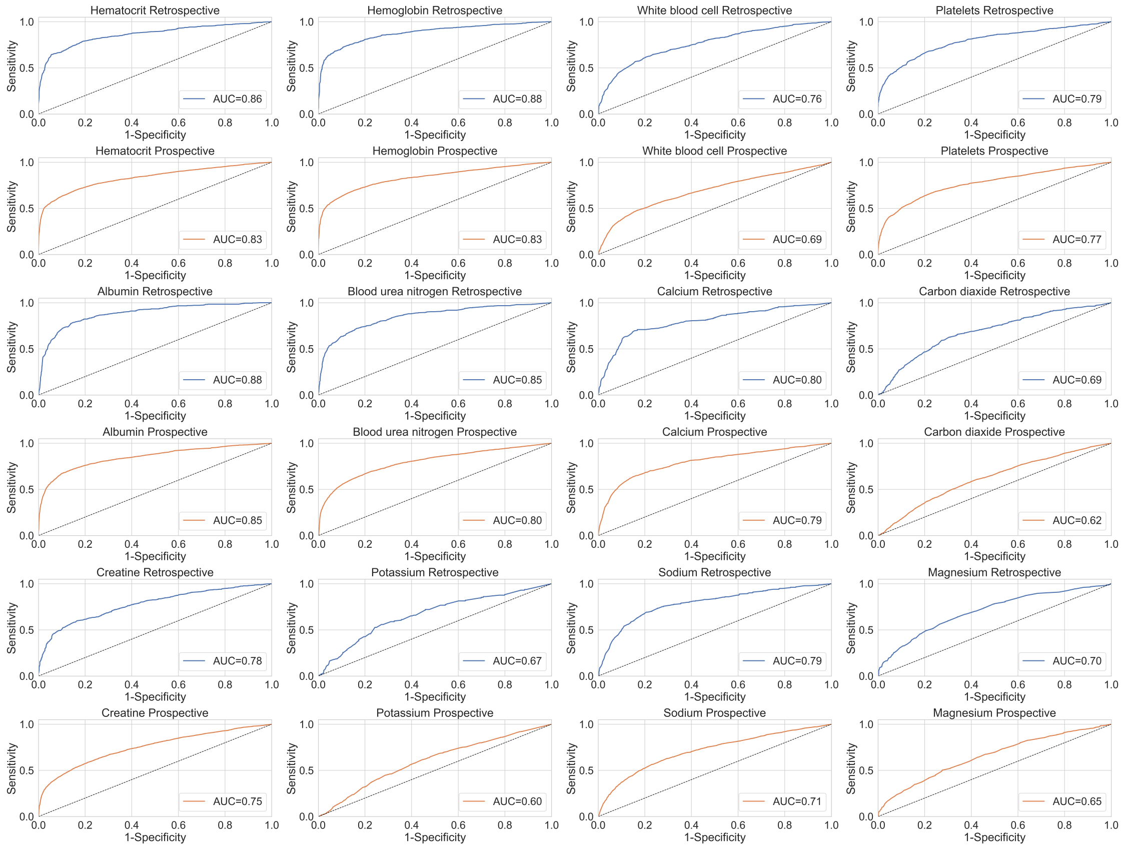

Supplementary Figure 1: Receiver operating characteristic curves for twelve deployed models on retrospective and prospective test sets.

Supplementary Table 2: Hemoglobin model performance by protected demographic groups

Prediction task	Group	Retrospective AUROC	Prospective AUROC
**Hemoglobin**	***Full cohort***	***0.88 [0.86, 0.89]***	***0.83 [0.83, 0.84]***
	sex_Female	0.88 [0.86, 0.90]	0.82 [0.81, 0.83]
	sex_Male	0.88 [0.86, 0.90]	0.84 [0.83, 0.85]
	race_Asian	0.87 [0.84, 0.90]	0.85 [0.84, 0.86]
	race_Black	0.85 [0.77, 0.93]	0.77 [0.74, 0.80]
	race_Native American	1.00 [1.00, 1.00]	0.81 [0.71, 0.90]
	race_Other	0.87 [0.84, 0.91]	0.82 [0.80, 0.83]
	race_Pacific Islander	0.85 [0.59, 1.00]	0.84 [0.80, 0.88]
	race_Unknown	0.80 [0.67, 0.90]	0.70 [0.63, 0.76]
	race_White	0.89 [0.87, 0.91]	0.84 [0.84, 0.85]
	age_over_40	0.89 [0.87, 0.91]	0.84 [0.83, 0.85]
	sex_Unknown	NaN	1.00 [1.00, 1.00]

Supplementary Table 3: White blood cell model performance by protected demographic groups

Prediction task	Group	Retrospective AUROC	Prospective AUROC
**White blood cell**	***Full cohort***	***0.76 [0.74, 0.79]***	***0.69 [0.68, 0.70]***
	sex_Female	0.77 [0.73, 0.80]	0.68 [0.67, 0.69]
	sex_Male	0.76 [0.73, 0.80]	0.70 [0.69, 0.71]
	race_Asian	0.76 [0.70, 0.82]	0.70 [0.68, 0.72]
	race_Black	0.75 [0.63, 0.86]	0.68 [0.64, 0.72]
	race_Native American	0.50 [0.13, 0.88]	0.68 [0.49, 0.82]
	race_Other	0.73 [0.68, 0.78]	0.67 [0.65, 0.69]
	race_Pacific Islander	1.00 [1.00, 1.00]	0.76 [0.69, 0.83]
	race_Unknown	0.87 [0.74, 0.96]	0.69 [0.60, 0.76]
	race_White	0.77 [0.73, 0.80]	0.70 [0.68, 0.71]
	age_over_40	0.76 [0.73, 0.79]	0.70 [0.69, 0.71]
	sex_Unknown	NaN	0.80 [0.40, 1.00]

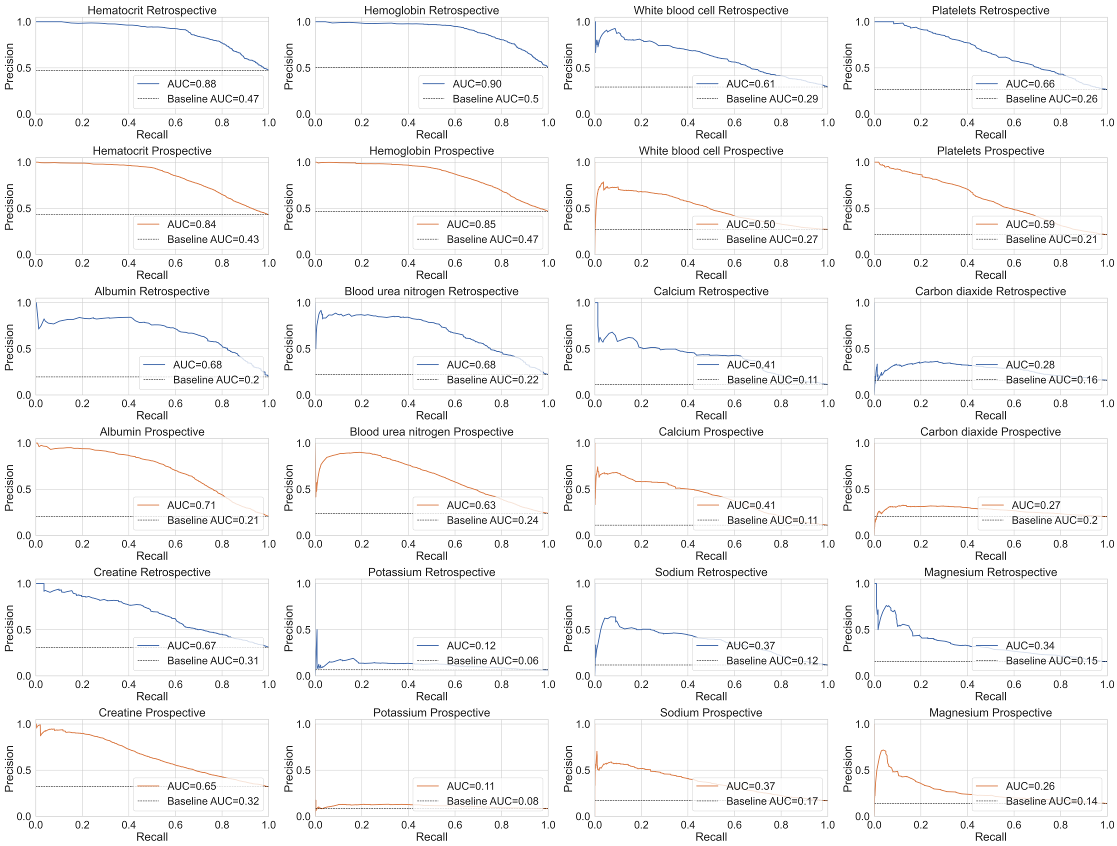

Supplementary Figure 2: Precision recall curves for twelve deployed models on retrospective and prospective test sets.

Supplementary Table 4: Platelets model performance by protected demographic groups

**Prediction task**	**Group**	**Retrospective AUROC**	**Prospective AUROC**
**Platelets**	***Full cohort***	***0.79 [0.77, 0.82]***	***0.77 [0.76, 0.78]***
	sex_Female	0.80 [0.77, 0.84]	0.76 [0.75, 0.77]
	sex_Male	0.78 [0.74, 0.81]	0.78 [0.77, 0.79]
	race_Asian	0.78 [0.72, 0.84]	0.79 [0.77, 0.81]
	race_Black	0.85 [0.75, 0.93]	0.76 [0.72, 0.79]
	race_Native American	0.89 [0.67, 1.00]	0.76 [0.60, 0.90]
	race_Other	0.76 [0.70, 0.81]	0.75 [0.73, 0.77]
	race_Pacific Islander	0.69 [0.07, 1.00]	0.77 [0.69, 0.85]
	race_Unknown	0.87 [0.72, 0.98]	0.64 [0.51, 0.74]
	race_White	0.80 [0.77, 0.83]	0.78 [0.76, 0.79]
	age_over_40	0.78 [0.75, 0.81]	0.77 [0.76, 0.78]
	sex_Unknown	NaN	NaN

Supplementary Table 5: Albumin model performance by protected demographic groups

**Prediction task**	**Group**	**Retrospective AUROC**	**Prospective AUROC**
**Albumin**	***Full cohort***	***0.88 [0.86, 0.91]***	***0.85 [0.84, 0.86]***
	sex_Female	0.89 [0.86, 0.91]	0.86 [0.85, 0.87]
	sex_Male	0.88 [0.86, 0.91]	0.84 [0.83, 0.85]
	race_Asian	0.81 [0.74, 0.87]	0.84 [0.82, 0.86]
	race_Black	0.91 [0.80, 0.97]	0.83 [0.78, 0.87]
	race_Native American	0.93 [0.61, 1.00]	0.80 [0.69, 0.90]
	race_Other	0.88 [0.83, 0.91]	0.84 [0.82, 0.86]
	race_Pacific Islander	0.73 [0.46, 0.96]	0.89 [0.83, 0.93]
	race_Unknown	0.84 [0.63, 1.00]	0.83 [0.75, 0.91]
	race_White	0.91 [0.89, 0.93]	0.86 [0.85, 0.87]
	age_over_40	0.89 [0.87, 0.91]	0.87 [0.86, 0.88]
	sex_Unknown	NaN	NaN

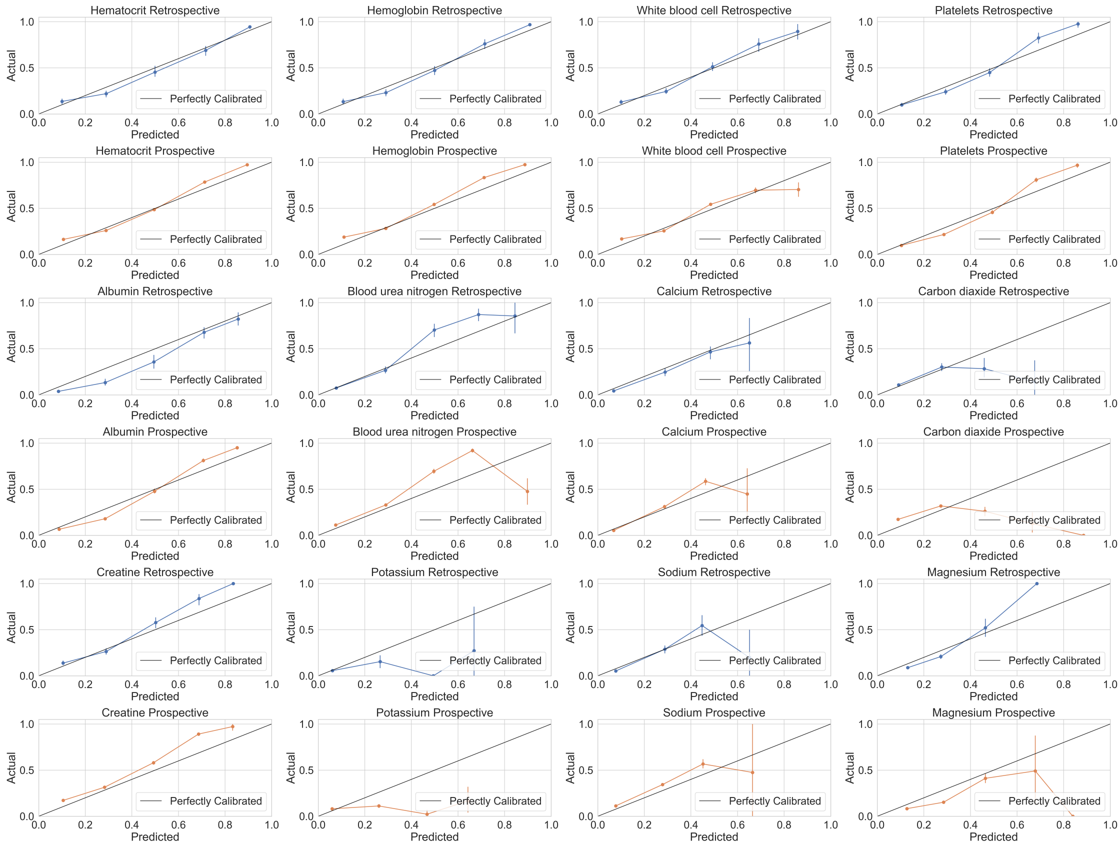

Supplementary Figure 3: Calibration plots for twelve deployed models on retrospective and prospective test sets.

Supplementary Table 6: Blood urea nitrogen model performance by protected demographic groups

**Prediction task**	**Group**	**Retrospective AUROC**	**Prospective AUROC**
**Blood urea nitrogen**	***Full cohort***	***0.85 [0.83, 0.87]***	***0.80 [0.79, 0.81]***
	sex_Female	0.84 [0.81, 0.88]	0.79 [0.77, 0.80]
	sex_Male	0.85 [0.82, 0.87]	0.80 [0.78, 0.81]
	race_Asian	0.86 [0.80, 0.92]	0.81 [0.79, 0.83]
	race_Black	0.85 [0.72, 0.95]	0.82 [0.78, 0.85]
	race_Native American	1.00 [1.00, 1.00]	0.91 [0.82, 0.97]
	race_Other	0.85 [0.80, 0.88]	0.78 [0.76, 0.80]
	race_Pacific Islander	0.91 [0.75, 1.00]	0.83 [0.77, 0.88]
	race_Unknown	0.85 [0.61, 0.99]	0.74 [0.67, 0.82]
	race_White	0.84 [0.81, 0.87]	0.79 [0.78, 0.80]
	age_over_40	0.85 [0.82, 0.87]	0.79 [0.78, 0.80]
	sex_Unknown	NaN	NaN

Supplementary Table 7: Calcium model performance by protected demographic groups

**Prediction task**	**Group**	**Retrospective AUROC**	**Prospective AUROC**
**Calcium**	***Full cohort***	***0.80 [0.76, 0.83]***	***0.79 [0.78, 0.81]***
	sex_Female	0.76 [0.70, 0.81]	0.78 [0.76, 0.80]
	sex_Male	0.83 [0.79, 0.87]	0.81 [0.79, 0.82]
	race_Asian	0.76 [0.66, 0.86]	0.78 [0.76, 0.81]
	race_Black	0.80 [0.69, 0.91]	0.75 [0.68, 0.81]
	race_Native American	0.78 [0.33, 1.00]	0.81 [0.68, 0.93]
	race_Other	0.84 [0.78, 0.89]	0.82 [0.80, 0.84]
	race_Pacific Islander	0.87 [0.73, 1.00]	0.81 [0.71, 0.89]
	race_Unknown	0.64 [0.36, 0.89]	0.78 [0.61, 0.93]
	race_White	0.78 [0.73, 0.84]	0.79 [0.77, 0.81]
	age_over_40	0.79 [0.75, 0.83]	0.79 [0.78, 0.81]
	sex_Unknown	NaN	NaN

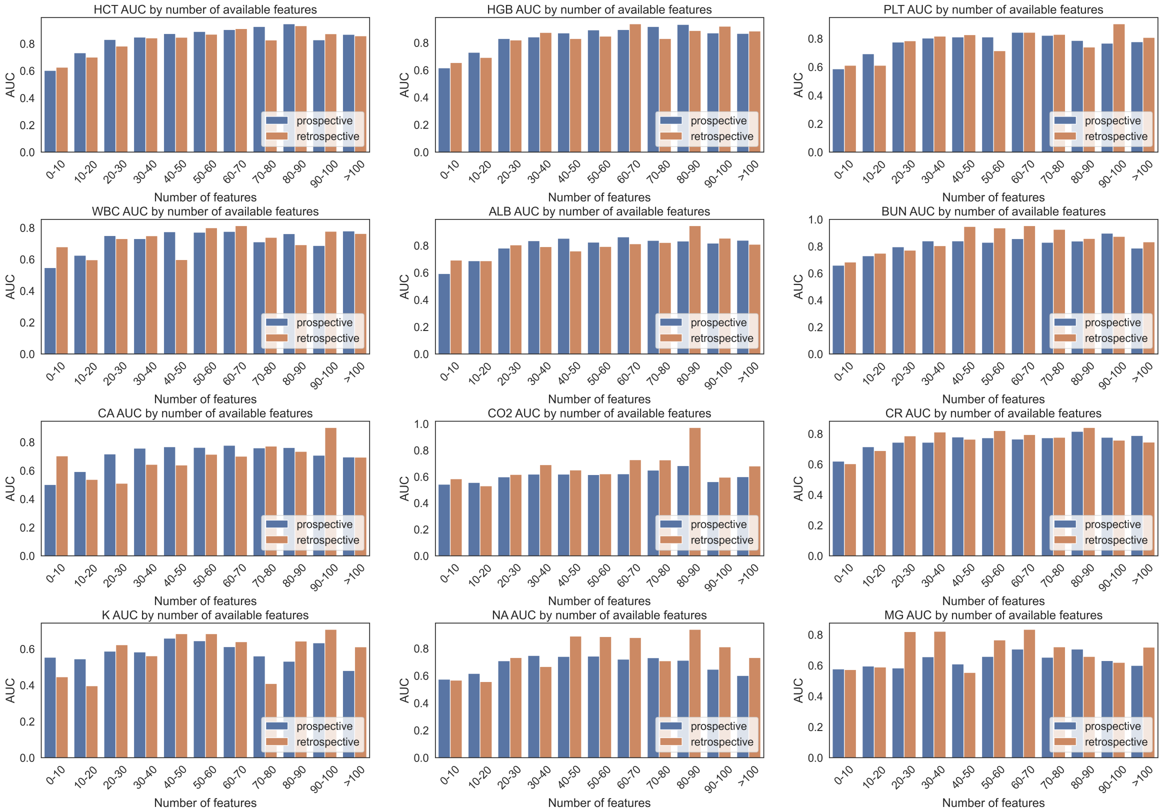

Supplementary Figure 4: AUC (area under the receiver operating characteristics curve) stratified by number of available features in retrospective and prospective test sets. AUC generally trends upwards as the number of available features increases. HCT=Hematocrit, HGB=Hemoglobin, PLT=Platelets, WBC=White Blood Cell Count, ALB=Albumin, BUN=Blood Urea Nitrogen, CA=Calcium, CO2=Carbon dioxide, CR=Creatinine, K=Potassium, NA=Sodium, MG=Magnesium.

Supplementary Table 8: Carbon dioxide model performance by protected demographic groups

**Prediction task**	**Group**	**Retrospective AUROC**	**Prospective AUROC**
**Carbon dioxide**	***Full cohort***	***0.69 [0.66, 0.72]***	***0.62 [0.61, 0.63]***
	sex_Female	0.67 [0.62, 0.71]	0.61 [0.59, 0.62]
	sex_Male	0.71 [0.66, 0.76]	0.63 [0.61, 0.64]
	race_Asian	0.73 [0.64, 0.80]	0.61 [0.59, 0.64]
	race_Black	0.76 [0.63, 0.88]	0.62 [0.58, 0.66]
	race_Native American	AUC undefined	0.74 [0.61, 0.84]
	race_Other	0.72 [0.65, 0.77]	0.63 [0.60, 0.65]
	race_Pacific Islander	0.26 [0.04, 0.54]	0.69 [0.62, 0.76]
	race_Unknown	0.96 [0.91, 1.00]	0.64 [0.55, 0.73]
	race_White	0.65 [0.60, 0.70]	0.61 [0.59, 0.62]
	age_over_40	0.69 [0.65, 0.72]	0.62 [0.61, 0.64]
	sex_Unknown	NaN	0.75 [0.25, 1.00]

Supplementary Table 9: Creatinine model performance by protected demographic groups

Prediction task	Group	Retrospective AUROC	Prospective AUROC
**Creatinine**	***Full cohort***	***0.78 [0.75, 0.80]***	***0.75 [0.74, 0.76]***
	sex_Female	0.76 [0.73, 0.80]	0.74 [0.73, 0.75]
	sex_Male	0.77 [0.74, 0.81]	0.75 [0.74, 0.76]
	race_Asian	0.79 [0.74, 0.85]	0.75 [0.73, 0.77]
	race_Black	0.85 [0.77, 0.93]	0.76 [0.73, 0.80]
	race_Native American	1.00 [1.00, 1.00]	0.93 [0.87, 0.98]
	race_Other	0.80 [0.75, 0.84]	0.72 [0.71, 0.74]
	race_Pacific Islander	0.67 [0.43, 0.88]	0.81 [0.75, 0.86]
	race_Unknown	0.62 [0.43, 0.80]	0.71 [0.64, 0.78]
	race_White	0.75 [0.72, 0.79]	0.75 [0.74, 0.76]
	age_over_40	0.78 [0.75, 0.80]	0.76 [0.75, 0.77]
	sex_Unknown	NaN	0.88 [0.50, 1.00]

Supplementary Table 10: Potassium model performance by protected demographic groups

Prediction task	Group	Retrospective AUROC	Prospective AUROC
**Potassium**	***Full cohort***	***0.67 [0.61, 0.72]***	***0.60 [0.59, 0.62]***
	sex_Female	0.65 [0.57, 0.72]	0.60 [0.58, 0.62]
	sex_Male	0.68 [0.62, 0.74]	0.60 [0.58, 0.63]
	race_Asian	0.75 [0.66, 0.83]	0.61 [0.57, 0.65]
	race_Black	0.79 [0.66, 0.90]	0.56 [0.50, 0.62]
	race_Native American	NaN	0.59 [0.46, 0.72]
	race_Other	0.64 [0.55, 0.74]	0.58 [0.55, 0.61]
	race_Pacific Islander	0.45 [0.06, 0.87]	0.56 [0.47, 0.64]
	race_Unknown	0.32 [0.10, 0.82]	0.64 [0.46, 0.80]
	race_White	0.65 [0.57, 0.72]	0.61 [0.58, 0.63]
	age_over_40	0.65 [0.59, 0.71]	0.60 [0.59, 0.62]
	sex_Unknown	NaN	0.00 [0.00, 0.00]

Supplementary Table 11: Sodium model performance by protected demographic groups

Prediction task	Group	Retrospective AUROC	Prospective AUROC
**Sodium**	***Full cohort***	***0.79 [0.75, 0.82]***	***0.71 [0.70, 0.72]***
	sex_Female	0.78 [0.73, 0.83]	0.71 [0.69, 0.72]
	sex_Male	0.79 [0.74, 0.83]	0.72 [0.70, 0.73]
	race_Asian	0.81 [0.73, 0.87]	0.73 [0.70, 0.75]
	race_Black	0.78 [0.65, 0.89]	0.71 [0.65, 0.75]
	race_Native American	0.73 [0.14, 1.00]	0.74 [0.59, 0.89]
	race_Other	0.78 [0.72, 0.84]	0.68 [0.65, 0.70]
	race_Pacific Islander	0.91 [0.81, 1.00]	0.70 [0.62, 0.78]
	race_Unknown	0.72 [0.45, 0.99]	0.75 [0.65, 0.84]
	race_White	0.78 [0.72, 0.83]	0.72 [0.71, 0.74]
	age_over_40	0.77 [0.73, 0.81]	0.71 [0.70, 0.72]
	sex_Unknown	NaN	0.20 [0.00, 0.60]

Supplementary Table 12: Magnesium model performance by protected demographic groups

Prediction task	Group	Retrospective AUROC	Prospective AUROC
**Magnesium**	***Full cohort***	***0.70 [0.67, 0.73]***	***0.65 [0.63, 0.67]***
	sex_Female	0.71 [0.66, 0.75]	0.67 [0.63, 0.70]
	sex_Male	0.70 [0.66, 0.74]	0.64 [0.61, 0.67]
	sex_Unknown	NaN	NaN
	race_Asian	0.76 [0.66, 0.84]	0.66 [0.61, 0.72]
	race_Black	0.69 [0.53, 0.82]	0.70 [0.62, 0.77]
	race_Native American	NaN	0.53 [0.29, 0.75]
	race_Other	0.69 [0.62, 0.74]	0.65 [0.61, 0.70]
	race_Pacific Islander	0.86 [0.67, 0.99]	0.60 [0.47, 0.72]
	race_Unknown	0.64 [0.28, 0.94]	0.42 [0.18, 0.67]
	race_White	0.70 [0.65, 0.74]	0.65 [0.62, 0.68]
	age_over_40	0.70 [0.66, 0.74]	0.65 [0.63, 0.68]
